# Elucidating immunologic mechanisms of PROSTVAC cancer immunotherapy

**DOI:** 10.1186/s40425-014-0034-0

**Published:** 2014-10-14

**Authors:** Stefanie J Mandl, Ryan B Rountree, Tracy B dela Cruz, Susan P Foy, Joseph J Cote, Evan J Gordon, Erica Trent, Alain Delcayre, Alex Franzusoff

**Affiliations:** Bavarian Nordic, Inc, 2425 Garcia Ave, Mountain View, CA 94043 USA; ExoThera LLC, 675 Olive Street, Menlo Park, CA 94025 USA

**Keywords:** PROSTVAC, Active immunotherapy, Prostate cancer, Mechanism of action, Immune correlates, Heterologous prime-boost, Teff :Treg ratio

## Abstract

**Background:**

PROSTVAC®, an active immunotherapy currently studied for the treatment of metastatic castration-resistant prostate cancer (mCRPC), consists of a heterologous prime-boost regimen with two different poxvirus-based vectors to provoke productive immune responses against prostate specific antigen (PSA) as the target tumor antigen. A Phase 2 study of PROSTVAC immunotherapy showed significantly improved median overall survival by 8.5 months and is currently being validated in a global Phase 3 study (PROSPECT; NCT01322490). Here, preclinical models were explored to investigate the mechanism of action and immune signatures of anti-tumor efficacy with PROSTVAC immunotherapy with the goal to identify potential immune correlates of clinical benefit.

**Methods:**

PROSTVAC-induced immune responses and anti-tumor efficacy were studied in male BALB/c mice. Functionality of the induced T cell response was characterized by interferon-gamma (IFNγ) ELISPOT, cytotoxic degranulation, multi-cytokine intracellular staining, and *in vivo* T cell depletion. Tumor infiltrating lymphocytes (TILs) were evaluated phenotypically by flow cytometry.

**Results:**

The heterologous prime-boost regimen of the two PROSTVAC vectors significantly enhanced the magnitude and quality of activated PSA-specific CD4 and CD8 T cell responses compared to homologous, single vector regimens. PROSTVAC-activated CD4 and CD8 T cells were highly functional as evidenced by expression of activation markers, production of multiple cytokines, and amplified cytotoxic T cell activity. Importantly, PROSTVAC immunotherapy resulted in significant anti-tumor efficacy in a transplantable prostate cancer mouse model. Antigen-spreading occurred in PROSTVAC-treated animals that rejected PSA-expressing tumors, as shown by subsequent rejection of PSA-negative tumors. *In vivo* CD4 and CD8 depletion revealed that both T cell subsets contributed to anti-tumor efficacy. Characterization of TILs demonstrated that PROSTVAC immunotherapy greatly increased the intra-tumoral ratio of activated effector to regulatory T cells.

**Conclusions:**

PROSTVAC immunotherapy activates broad, highly functional T cell immunity to PSA and to endogenous tumor antigens via immune-mediated antigen spreading. These preclinical results further elucidate the mode of action of PROSTVAC immunotherapy and its potential causal relationship to extended overall survival as observed in the PROSTVAC Phase 2 study. The clinical validation is ongoing in the PROSPECT Phase 3 clinical study.

**Electronic supplementary material:**

The online version of this article (doi:10.1186/s40425-014-0034-0) contains supplementary material, which is available to authorized users.

## Background

PROSTVAC® is a PSA-targeted active immunotherapy composed of a heterologous prime-boost regimen using two poxviral-based vectors: PROSTVAC-V™, a recombinant vaccinia virus, and PROSTVAC-F™, a recombinant fowlpox virus. Both vectors contain transgenes for human prostate-specific antigen (PSA) and three co-stimulatory molecules for T cells (B7.1, ICAM-1, and LFA-3, designated as TRICOM) to enhance immune activation [1]. A robust data package for PROSTVAC has been generated through 8 completed Phase 1 and Phase 2 clinical trials, where more than 300 patients have been treated. The PROSTVAC immunotherapy regimen is well-tolerated, with grade 2 or less injection site reactions being most common [2]. A randomized, placebo-controlled Phase 2 study in patients with asymptomatic or minimally symptomatic mCRPC used the heterologous prime-boost strategy composed of a single PROSTVAC-V priming dose followed by six PROSTVAC-F boosts. All virus treatments were given in combination with low-dose recombinant GM-CSF. This study demonstrated the potential ability of PROSTVAC to extend the median overall survival by 8.5 months in patients with advanced prostate cancer (25.1 months vs. 16.6 months for controls) and reduced the death rate by 44% [3]. By comparison, the clinical studies supporting FDA approval of sipuleucel-T for mCRPC improved the median overall survival by 4.1 months [4]. An international Phase 3 study (PROSPECT) is currently ongoing to validate the randomized Phase 2 data with PROSTVAC for the treatment of men with mCRPC. Concurrently, PROSTVAC is being investigated in NCI-sponsored clinical studies in different stages of prostate cancer and in combination with other anticancer agents, such as anti-androgens, local radiation, targeted small molecule drugs, and immune checkpoint inhibitors [5].

Throughout the various Phase 1 and Phase 2 studies performed with PROSTVAC and its precursors, immune monitoring has been performed and the collective data on the immune impact of PROSTVAC has recently been published by Gulley et al [2]. For example, findings of a small Phase 2 study (n = 32) investigating the influence of immunologic and prognostic factors on overall survival suggested that patients who mounted the highest increase (>6 fold as compared to baseline) in PSA-specific T cell responses pre and post PROSTVAC treatment, showed improved survival compared with patients that did not mount as great a response [6]. Additionally, patients surviving longer than predicted by Halabi nomogram showed a decrease in the suppressive function of CD4 T regulatory (Treg) cells while it was increased in patients surviving shorter than predicted. Overall, this study suggested that patients with more indolent mCRPC (Halabi predicted survival >18 months) may best benefit from PROSTVAC immunotherapy [2,6,7].

The data for potential immune correlates of improved clinical benefit, while revealing and hypothesis-generating for prospective analyses, were generated retrospectively from a small subset of all subjects. Moreover, most ELISPOT assays to date used a single 9-mer peptide representing only the T cell responses of patients with the HLA-A2 allele, and tumor tissue for the evaluation of tumor-infiltrating immune responses after subcutaneous dosing with PROSTVAC was not available. Hence, more work is needed to establish a correlation between immune responses and improved overall survival.

PROSTVAC-V/F immunotherapy is a complex biologic that has been developed in an iterative fashion in parallel with other vectors expressing CEA as tumor associated antigens. Using vectors that express human CEA and mouse TRICOM molecules in the context of CEA-transgenic mice it has been demonstrated that poxvirus-based immunotherapy can overcome tolerance and result in significant anti-tumor efficacy against transplanted and spontaneously arising tumors [8-10].

The data presented here employed the PROSTVAC-V/F heterologous prime-boost regimen in WT BALB/c mice, which allowed for an in-depth characterization of peripheral and tumor-infiltrating T cell responses induced by the PROSTVAC immunotherapy for the first time. Our data not only support the previous clinical immunological data, but also provide additional mechanistic evidence that has been lacking from clinical studies. PROSTVAC immunotherapy induced polyfunctional PSA-specific T cells, resulted in the infiltration of T cells into tumors, required CD8 T cells for anti-tumor efficacy, and led to antigen spreading which protected mice from re-challenge with tumor cells that lacked PSA expression. Our data provide insight towards prospective and retrospective analyses exploring the role of PROSTVAC immunotherapy for improved clinical benefit.

## Results

### Heterologous prime-boost strategy increases the magnitude and quality of PSA-specific CD4 and CD8 T cell responses

**Figure 1:**
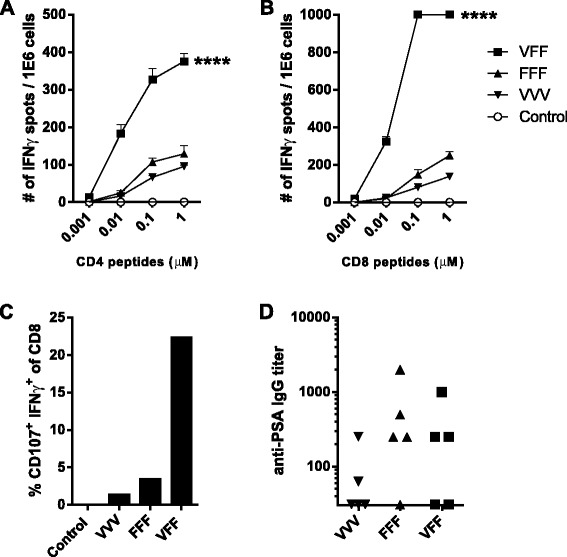
**Heterologous prime-boost amplifies PSA-specific T cell responses.** BALB/c males (5/group) were treated every two weeks with: buffer (Control), PROSTVAC-V (VVV), PROSTVAC-F (FFF) or received a PROSTVAC-V prime followed by 2 PROSTVAC-F boosts (VFF). Pooled splenocytes were assayed for PSA-specific responses by IFNγ ELISPOT **(A, B)** and cytotoxic activity by flow cytometry (% CD107^+^ IFNγ^+^ CD8 T cells) **(C)**. Anti-PSA IgG titers were determined by ELISA for each individual mouse **(D)**. For ELISPOT, splenocytes were restimulated with CD4 or CD8 PSA-specific peptides or controls (controls not shown) at indicated concentrations. Responses that were too numerous to count were displayed as 1000 spots/million cells. Statistical significance was determined by Two-way-ANOVA with Tukey post-test at 0.01 μM. ****P < 0.001 compared to control or homologous dosing **(A** & **B)**. To identify cytotoxic CD8+ T cells, splenocytes were restimulated overnight with a PSA CD8-specific peptide in the presence of anti-CD107 antibody. Graphs show representative data of four independently performed experiments.

The PROSTVAC-V/F dosing regimen in the PROSPECT Phase 3 study comprises a heterologous prime-boost regimen consisting of a single prime with PROSTVAC-V followed by six consecutive doses of PROSTVAC-F [3]. The regimens in mouse studies described here were performed with a maximum of three homologous vector doses or one priming plus two heterologous vector doses. Male BALB/c mice were immunized three times with PROSTVAC-V (VVV), three times with PROSTVAC-F (FFF), or were primed with PROSTVAC-V, followed by booster doses of PROSTVAC-F (VFF). Two weeks after the last dose, PSA-specific immune responses were evaluated by IFNγ ELISPOT, CTL degranulation assay, and ELISA (Figure 1; for comparison, vector-specific responses can be found in the Additional file 1: Figure SD1).

**Figure 2:**
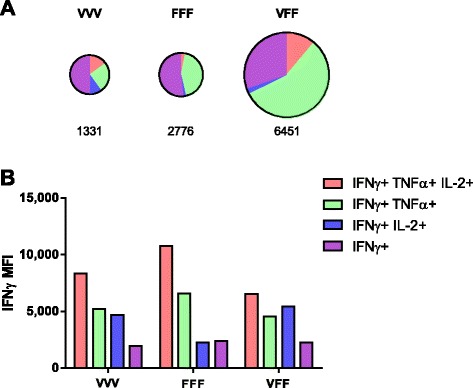
**Heterologous prime-boost improves the quality of PSA-specific T cell responses.** BALB/c mice (6/group) were treated as described for Figure 1. Spleens were harvested 14 days after the last treatment, and pooled splenocytes were restimulated overnight with PSA OPL or controls (controls not shown). The cells were stained for intracellular IFNγ, TNFα, and IL-2 prior to flow cytometric analysis. **(A)** The pie charts are weighted in size to reflect the numbers of detected cells (total numbers of PSA-specific CD8 per million T cells are indicated below each chart, % of respective T cell populations can be found in Additional file 1: Table SD1). **(B)** Amount of IFNγ production on a per cell basis as measured by mean fluorescence intensity (MFI). Graphs show representative data of two independently performed experiments.

The VFF heterologous prime-boost regimen resulted in a much higher frequency of IFNγ-producing PSA-specific CD4 T cells (Figure 1A) and CD8 T cells (Figure 1B and Figure 2A) compared to VVV or FFF homologous dosing regimens. Moreover, PSA-specific T cells from VFF dosing were of higher avidity (Figure 1A & B), as evidenced by higher frequencies of T cells responding at the lower 0.01 μM peptide concentrations in the ELISPOT. Importantly, the number of functionally active PSA-specific CD8 CTLs resulting from the VFF heterologous prime-boost regimen was 7 to 20 fold higher than those generated by either homologous dosing regimen (Figure 1C). In contrast to the T cell responses, the heterologous prime-boost regimen did not improve PSA-specific antibody responses (Figure 1D). These results indicate that heterologous VFF dosing generates CD4 and CD8 PSA-specific T cell responses of greater magnitude and higher quality as measured by higher avidity and increased CD8 CTL activity. These traits would contribute to improved anti-PSA specific anti-tumor responses following heterologous PROSTVAC-V/F dosing.

Additional distinguishing features in the quality of the PSA-specific CD8 T cell response were observed when PSA-specific CD8 T cells were analyzed for the multicytokine-production of IFNγ, TNFα, and IL-2 by flow cytometry (Figure 2). Using cytokine expression, CD8 memory T cells have been classified as double-positive CD8 T cells (IFNγ + TNFα+, represented in green) and as triple-positive CD8 T cells (IFNγ + TNFα + IL-2+, represented in red) [11]. In addition to the increased magnitude of the CD8 T cell response (Figure 1 and Figure 2A), a pronounced shift in the quality of the CD8 T cell response was revealed. Particularly a higher proportion of double-positive T cells (56% for VFF as compared to 25% (VVV) and 43% (FFF)) resulted from the heterologous PROSTVAC-V/F regimen compared to homologous dosing regimen (Figure 2A and Additional file 1: Table SD1). The percent of triple-positive PSA-specific T cells was similar in the VVV and VFF groups but higher as compared to FFF treatment. Priming with a 5 fold higher PROSTVAC-V dose did not yield any additional benefit in the magnitude or the quality of the CD8 T cell response (see Additional file 1: Figure SD2 and Table SD1). The increased production of IFNγ by multicytokine-producing cells has been linked to better protection against various infectious diseases [12]. Indeed, double-positive and triple-positive PSA-specific CD8 T cells produced higher levels of IFNγ on a per cell basis than single positive cells (Figure 2B). This increased IFNγ production was observed in double and triple positive CD8 T cells regardless of dosing regimen.

### Heterologous prime-boost regimen focuses the CD8 effector response towards the tumor antigen

**Figure 3:**
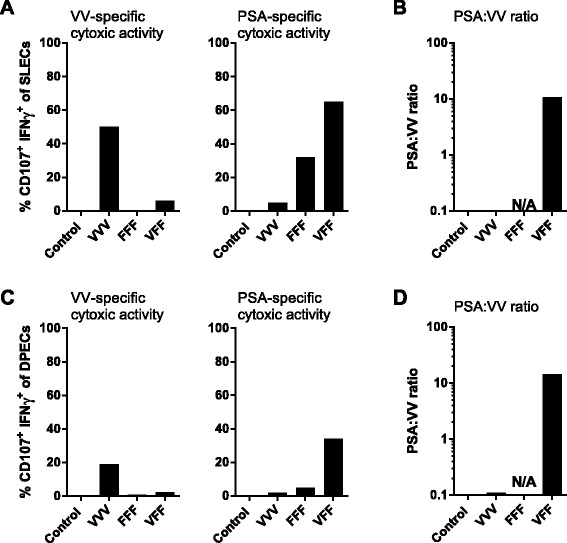
**Immune focusing of T cell response towards PSA.** Mice were treated as described for Figure 1. Pooled splenocytes were assayed for vaccinia virus (VV)-specific (**A** and **C** panels on left) or PSA-specific (**A** and **C** panels on right) cytotoxic activity by flow cytometry (% CD107+ IFNγ + CD8 T cells) 14 days after the last treatment. Splenocytes were restimulated overnight with vaccinia E3L and F2L peptides or with PSA OPL (or PSA peptide HPQKVTKFML, see Additional file 1: Figure SD4) in the presence of anti-CD107 antibody. The following day, cells were stained intracellularly for IFNγ and with the surface markers CD127 and KLRG1.% antigen-specific cytotoxic SLEC and DPEC were determined by gating on (CD8 + CD127-KLRG1+) and (CD8 + CD127 + KLRG1+) cells, respectively. **(B, D)**The PSA:VV ratio was determined by dividing % CD107+ IFNγ + PSA-specific DPECs or SLECs by % CD107+ IFNγ + VV-specific DPECs or SLECs. Graphs show representative data of two independently performed experiments. Similar responses were observed when splenocytes were restimulated with PSA peptide HPQKVTKFML instead of a PSA OPL (Additional file 1: Figure SD4). The gating strategy for these experiments is outlined in Additional file 1: Figure SD3).

Two additional T cell markers are regularly used to characterize highly functional CD8 effector T cell subsets, the CD127 (IL-7R alpha chain) memory T cell marker and the effector T cell marker killer lectin-like receptor G1 (KLRG-1) [13]. Short-lived effector CD8 T cells (SLECs) are characterized as KLRG-1+ CD127- CD8 T cells while double positive effector cells (DPECs) are characterized as KLRG1+ CD127+ CD8 T cells [14]. DPECs have recently been identified as a highly functional effector-like memory T cell subset [15]. The impact of heterologous PROSTVAC-V/F dosing compared to homologous dosing on the cytotoxic capabilities of vector-specific vs. PSA-specific effector T cell subsets was analyzed by flow cytometry. Homologous VVV dosing generated a relatively high number of vaccinia-specific cytotoxic SLEC (~50%) and DPEC (~20%) (Figures 3A, 3C), yet less than 10% of SLEC or DPEC cytotoxic CD8 T cells were PSA-specific. Conversely, 65% of SLEC and 30% of the highly active DPEC effector memory T cells were PSA-specific CTL following heterologous VFF dosing, while less than 10% constituted vaccinia-specific CTL (Figure 3A, 3C). Therefore, the heterologous PROSTVAC-V/F regimen resulted in a 100 fold improvement in the ratio of PSA-targeted to vaccinia-targeted SLEC and DPEC T cell responses (Figures 3B and D). Again, priming with 5 fold more PROSTVAC-V did not yield any additional benefit (data not shown).

The data shown in Figures 1, 2 and 3 revealed that the heterologous PROSTVAC-V/F regimen greatly enhances the magnitude and quality of the PSA-specific T cell response compared to homologous dosing with the same vector. Of note, a distinctive phenotype of activated, highly functional SLEC and DPEC was induced while priming with PROSTVAC-V and boosting with PROSTVAC-F provided the added benefit of focusing the highly functional CD8 CTL immune response towards PSA, the target tumor antigen, and away from the vaccinia vector.

### Heterologous PROSTVAC-V/F dosing induces significant anti-tumor efficacy and antigen-spreading in a mouse prostate cancer model

**Figure 4:**
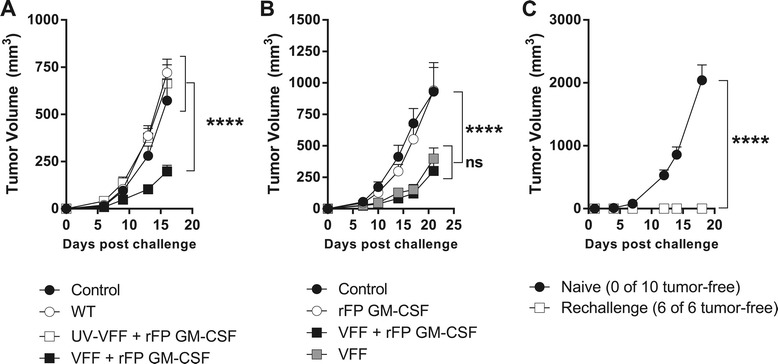
**PROSTVAC mediated anti-tumor efficacy and antigen spreading. (A **&** B)** BALB/c mice (10-20/group) were challenged by i.d. injection of RM-11-PSA cells on day 1 and treated 3 times at weekly intervals as described in text. Graphs show data from 2 independently performed experiments. **(C)** Previously-challenged, tumor-free PROSTVAC V/F-treated mice (n = 6) and naïve BALB/c (n = 10) were injected i.d. with 2E5 RM11-WT cells. ****P < 0.0001.

The anti-tumor activity of heterologous PROSTVAC-V/F immunotherapy was confirmed in a mouse prostate cancer model using transplanted RM-11-PSA cells. Mice were challenged with tumors and subsequently dosed with a regimen of heterologous wild-type (WT) parental virus vectors with no transgenes, with heterologous UV-inactivated PROSTVAC-V/F or heterologous PROSTVAC-V/F active immunotherapy (Figures 4A). The dosing regimen was accelerated to weekly administration due to the rapid growth kinetics of this tumor model. The anti-tumor response was dependent on dosing with active viral vectors, since no efficacy was observed with UV-inactivated viruses (Figure 4A). Although a visible trend in anti-tumor activity was noted within a 100 fold PROSTVAC-V/F dose range (Additional file 1: Figure SD5), the efficacy of PROSTVAC-V/F immunotherapy, compared to dosing with empty vectors (2E7/1E8 WT controls), was statistically equivalent over that 100 fold dose range. For all follow on studies, the highest dose of 2E7/1E8 VFF was used.

In Figure 4A, all dosing was accompanied by immunization with 1E7 Inf.U of recombinant fowlpox virus encoding murine GM-CSF (rFP GM-CSF). This dose has been shown to have similar activity as recombinant GM-CSF in mouse models [16]. Recombinant GM-CSF was used in the Phase 2 clinical trial that resulted in improved median overall survival of 8.5 month in men with mCRPC [3]. However, the necessity of GM-CSF for the efficacy of PROSTVAC immunotherapy remains to be determined. A comparison of PROSTVAC-V/F immunotherapy with and without rFP GM-CSF is shown in Figure 4B. Anti-tumor efficacy was observed whether or not murine GM-CSF was co-administered with PROSTVAC-V/F (Figure 4B). Treatment with the rFP GM-CSF vector alone had no effect on tumor growth.

As shown in Figure 4A, the anti-tumor efficacy in the RM-11-PSA model was unequivocally dependent on a productive immune response against the vector-encoded PSA tumor antigen compared to WT parent vectors. The requirement for a response to the delivered tumor antigen highlights the contribution of the adaptive immune response to anti-tumor efficacy. Successive induction of T cell immune responses against endogenous tumor antigens not encoded by the vector (antigen spreading) has gained attention as a crucial component for effective, long-lasting tumor surveillance in addition to the transgene specific anti-tumor activity [17,18]. To test if adaptive immune responses initiated by PROSTVAC therapy had spread to other antigens, mice were implanted with RM-11-PSA tumor cells and treated with PROSTVAC or control buffer as described before. Six out of 20 mice completely rejected the tumor (data of individual mice of this experiment are shown in Additional file 1: Figure SD6). Animals that rejected PSA-expressing tumors were subsequently re-challenged with a lethal dose of parental RM-11-WT tumors that lack PSA expression. All re-challenged mice completely rejected the parental tumors while naïve mice challenged with the same parental tumor cells succumbed within 18 days (Figure 4C). These data confirm that PROSTVAC-V/F immunotherapy induced antigen-spreading as a consequence of active anti-cancer PSA-specific T cell responses. While the target antigens of the T cells that contributed to the *de novo* anti-tumor efficacy were not determined, these results demonstrated that the protective immunity elicited by PROSTVAC immunotherapy expanded to include antigen-spreading beyond the robust PSA-specific T cell immune responses identified in Figures 1, 2 and 3.

### Tumor infiltrating PSA-specific CD8 and CD4 T lymphocytes contribute to anti-tumor efficacy

**Figure 5:**
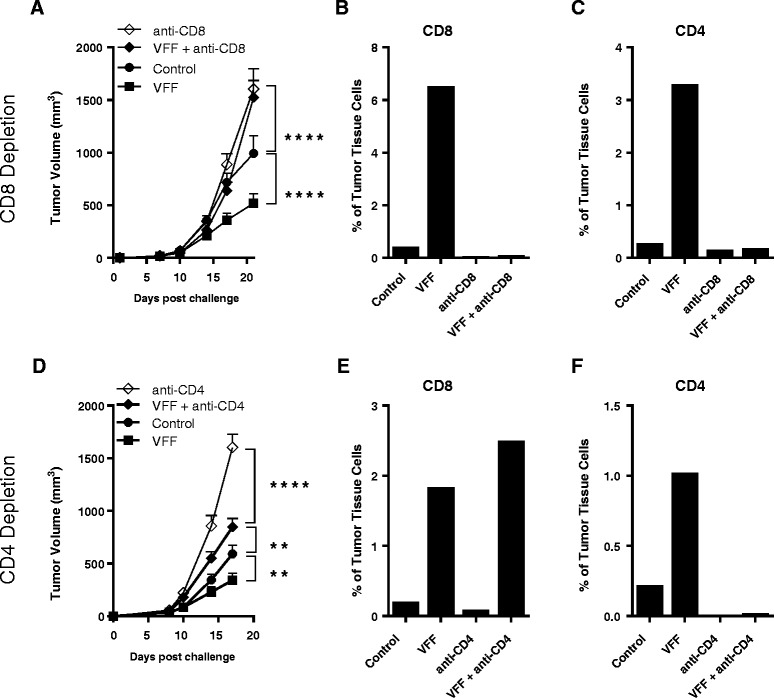
**Anti-tumor efficacy in CD4 or CD8 T cell depleted mice and analysis of TILs.** Selected groups of BALB/c mice (10/group) were injected i.p. with either anti-CD8 **(A)** or anti-CD4 antibodies **(D)** prior to tumor cell challenge and throughout the study. Mice were treated 3 times at weekly intervals starting on day 1. **P < 0.01, ****P < 0.0001 as compared to control. At the end of the study, tumors were removed and TILs were analysed by flow cytometry. CD8 **(B** and **E)** and CD4 **(C** and **F)** T cells are shown as a percentage of cells isolated from the tumors. Graphs show representative data from two independently performed experiments.

The infiltration of highly functional antigen-specific CTLs into the tumor coupled with overcoming the regulatory CD4 T cell environment in the tumor are considered to be key drivers for optimal anti-tumor efficacy [19]. To explore the contributions of CD4 and CD8 TILs to anti-tumor efficacy, heterologous PROSTVAC-V/F immunotherapy was performed in mice that were selectively depleted of CD8 T cells (Figure 5A) or CD4 T cells (Figure 5D). Tumors were subsequently isolated and the phenotype of TILs was analyzed by flow cytometry (Figure 5B and C for CD8 depletion and Figure 5E and F for CD4 depletion).

As shown before (Figure 4A and B), heterologous PROSTVAC immunotherapy resulted in significant delay of tumor growth as compared to control-treated animals (Figure 5A and 5D). This anti-tumor efficacy was characterized by the infiltration of the tumor by CD8 T cells (see VFF, Figure 5B and 5E) and CD4 T cells (see VFF, Figure 5C and 5F) at approximately a 2:1 ratio of CD8 to CD4 T cells. The depletion of CD8 T cells completely abrogated PROSTVAC-mediated anti-tumor efficacy, demonstrating the importance of PROSTVAC-induced CD8 effector cells. As expected, no CD8 T cells were found in CD8-depleted animals (Figure 5B), and this may explain why tumors grew more aggressively at later time points in these animals compared to controls. In addition, fewer CD4 T cells infiltrated the tumor of CD8-depleted animals (Figure 5C), suggesting that CD8 T cells aid in the recruitment of CD4 T cells into the tumor.

CD4 T cell depletion alone also had an impact on tumor growth and significantly increased the aggressiveness of the tumor model in the absence of PROSTVAC treatment. TIL analysis showed that no CD4 and very few CD8 T cells could be found in tumors of CD4-depleted animals (Figure 5E and F). Importantly, the impact of CD4 depletion was partially overcome by PROSTVAC immunotherapy (Figure 5D). Albeit not as effective as in fully immune-competent animals, PROSTVAC immunotherapy in the absence of CD4 T cells significantly reduced tumor growth as compared to CD4-depleted animals (VFF + anti-CD4 vs. anti-CD4). Subsequent TIL analysis revealed that PROSTVAC dosing had, even in the absence of CD4 T cells, recruited CD8 effector T cells into the tumor suggesting that PROSTVAC immunotherapy activated and recruited CD8 T cell infiltration independently of CD4 T cell help (VFF + anti-CD4, Figure 5E). These findings suggest that PROSTVAC immunotherapy allows for CD4-dependent and CD4-independent CD8 effector T cell generation.

### PROSTVAC immunotherapy recruits highly functional effector T cells into the tumor, while overcoming the tumor-associated Treg environment

The robust infiltration of CD4 and CD8 T cells into the tumor following PROSTVAC immunotherapy is critical for productive anti-cancer immunity. However, the presence of Treg cells in the tumor environment is recognized to hamper the development of effective anti-tumor responses [19-23].

**Figure 6:**
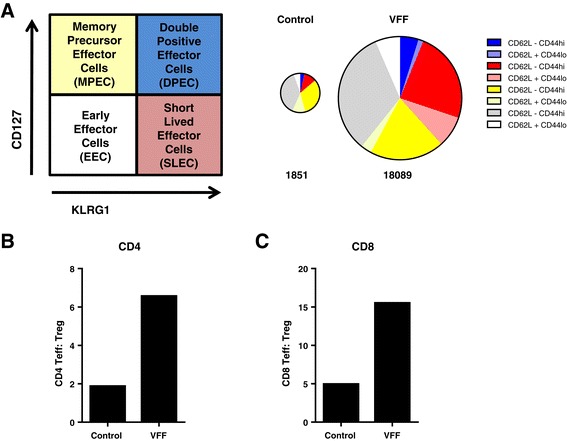
**PROSTVAC immunotherapy expands highly activated CD8 effector T cells in the tumor and improves the T**_**eff**_ **:T**_**reg**_ **cell ratios. (A)** Schematic for the characterization of effector and memory cells. Pie charts are weighted in size to reflect the numbers of detected cells (numbers below indicate total number of CD8 T cells/million tumor cells). **(B)** CD4 and **(C)** CD8 Teff :Treg ratios were calculated by dividing the number of CD4+ FoxP3- or CD8+ T cells by the number of CD4+ FoxP3+ Treg cells. Graphs show representative data from four independently performed experiments.

To confirm that PROSTVAC immunotherapy induced a productive and functional tumor-infiltrating T cell response, CD8 and CD4 TILs were analyzed for effector and memory T cell markers (KLRG-1 and CD127) and the CD62L^‒^ CD44^hi^ activation phenotype. Alternatively, ICOS was used as a marker for T cell activation (Additional file 1: Figure SD7). As shown in Figure 6A, the magnitude of CD8 TILs increased 10 fold (from 1851 to 18089 CD8 T cells/per million cells) in PROSTVAC-treated animals as compared to control-treated animals, indicating that PROSTVAC immunotherapy triggered a robust infiltration of CD8 T cells into the tumor. In addition to increasing the magnitude, the quality of the TIL response was greatly impacted by PROSTVAC immunotherapy. The CD8 TIL population in PROSTVAC-treated mice comprised a much higher proportion of SLECs (Figure 6A in red) and the highly functional effector memory DPECs (Figure 6A in blue). The majority of these cells also displayed the CD62L^‒^ CD44^hi^ activation phenotype. In contrast, the CD8 TIL population in control animals was composed mostly of early effector cells (EECs, CD127- KLRG-1-, Figure 6A in grey) and memory precursor effector cells (MPECs, CD127+ KLRG-1-, Figure 6A in yellow).

CD4 TILs were also analyzed for the Treg marker FoxP3. CD4 TIL phenotyping revealed that the frequency of CD4+ FoxP3+ Treg cells was reduced 2.5-fold, from an average of 31% of CD4 TILs in control animals to an average of 12% of CD4 TILs in PROSTVAC-V/F-treated animals (data not shown). A similar trend was also observed when ICOS was used as an additional marker on Treg (30% in controls vs. 12% in treated animals, Additional file 1: Figure SD7 and data not shown). Since the highly functional TILs resulting from PROSTVAC immunotherapy greatly outnumbered the tumor-associated regulatory CD4 Treg population, PROSTVAC immunotherapy considerably increased the ratios of CD4 and CD8 effector T cells to suppressive Treg cells (Teff : Treg ratio) in the tumors (Figure 6B and C). These data demonstrate that PROSTVAC immunotherapy improves the intratumoral balance of effector to regulatory T cells. Together these data reveal the mechanisms of PROSTVAC immunotherapy for eliciting significant, highly productive anti-tumor immune responses.

## Discussion

The preclinical studies in this work interrogated the immune signature generated by the heterologous prime-boost regimen and the mechanism of action of PROSTVAC immunotherapy in preclinical mouse models. We used a combination of functional assays and flow cytometry to characterize PSA-specific T cell responses induced by PROSTVAC treatment. Our data show that PROSTVAC treatment induced polyfunctional PSA-specific T cell responses as measured by IFNγ ELISPOT, multicytokine production and PSA-specific cytotoxic activity. The heterologous prime-boost regimen increased the magnitude of the PSA-specific T cell response as well as markedly changing the quality of the T cell response. Animals that received the heterologous prime-boost regimen showed higher percentages of IFNγ TNFα double -positive or IFNγ TNFα IL-2 triple-positive CD8 T cells. These cells have been described by Seder et al as double-positive effector memory (T_EM_) cells or triple-positive central memory (T_CM_) cells [11,24]. Besides the expression of IL-2, additional markers typically distinguish these T cell subsets. T_CM_ have a high proliferative potential, recirculate preferentially through lymph nodes, and require a relatively longer time to become cytotoxic. T_EM_, in contrast, have a less proliferative potential, recirculate preferentially through non-lymphoid tissues, and are immediately cytotoxic upon antigen re-exposure. Both T cell memory subsets have been reported to contribute effectively to protection from infectious disease, depending on the route, dose, replication rate and tropism of the infectious challenge [25]. Less is known about the relative contributions of these T cell subsets to productive immunity against cancer, but it is feasible to assume that polyfunctional CD8 T cells will prove to be more efficient in cancer immunotherapy. Higher percentages of CD8 T cells in the tumor have been correlated with better prognosis for a variety of cancers [26,27].

CD8 T cells phenotyped by CD127 and KLRG-1 biomarkers showed that poxvirus-based immunotherapy potently induces peripheral and tumor-infiltrating SLECs and DPECs which are activated effector cells with cytotoxic activity [28]. Although limitations in cell number prevented us from performing functional assays on the TILs, we believe that the phenotypic similarities of these highly activated (CD44+ and ICOS+) CD8 T cells add suggestive evidence that these cells have similar functional capabilities as the cells described in Figures 1, 2 and 3. Cytotoxic activity of TIL has been demonstrated by us in a different tumor model using a similar poxvirus-based immunotherapy product [29]. It is of note that the heterologous PROSTVAC prime-boost regimen increases the ratio of PSA-targeted to vaccinia-targeted CTL responses more than 100 fold compared to a homologous dosing regimen. These data are consistent with and may help explain the results from a Phase 2 clinical study investigating PROSTVAC dosing regimens. In that study, progression-free clinical benefit was much better in the cohort of men administered PROSTVAC-V as a priming dose followed by boosting with subsequent PROSTVAC-F doses (VFFF) over other regimens of PROSTVAC-F followed by PROSTVAC-V (FFFV), or homologous repeated FFFF doses with PROSTVAC-F [30].

Anti-tumor efficacy in the mouse model described here was dependent on live virus and the expression of PSA, but not on the co-administration of GM-CSF. Although low-dose recombinant GM-CSF was co-administered with PROSTVAC in the Phase 2 study showing improved overall survival in men with mCRPC, it is unclear what benefit was contributed by the addition of GM-CSF. Data from a Phase 2 study similar to that shown in Figure 4B also indicate that the clinical benefit for men with mCRPC treated with PROSTVAC may not be dependent on the co-administration with GM-CSF [6]. To test this hypothesis, the PROSPECT Phase 3 trial comprises two PROSTVAC treatment arms, i.e. with and without concomitant GM-CSF.

The use of a mouse model allowed us to investigate the mechanism of action of the observed anti-tumor activity more deeply by enabling *in vivo* depletion of T cell subsets and examination of TILs. *In vivo* depletions of CD8 and CD4 T cells confirmed that CD8 T cells are required and CD4 T cells are necessary for optimal anti-tumor efficacy. Importantly, it also demonstrated that CD4-dependent and independent mechanisms for the generation of CD8 T cells may exist. Phenotypic analysis of TILs demonstrated that the endogenous immune response to the tumor is weak in this model, although a low percentage CD4 and CD8 T cells could be detected. PROSTVAC immunotherapy, in contrast, increased tumor infiltration by CD8 and CD4 T cells more than 10-fold. More importantly, SLECs and DPECs were found in the tumor and these highly functional TILs greatly outnumbered the tumor-associated regulatory CD4 Treg population, resulting in much improved Teff : Treg ratios in the tumor microenvironment. Although PSA is a foreign antigen in the mice used here, the results found can be expected to apply towards tolerant transgenic mice where poxviral immunotherapy has already been demonstrated to be effective, despite having a lower magnitude response in transgenic compared to wild-type mice. Anti-tumor efficacy of poxvirus-based immunotherapy against an immune tolerant tumor associated antigen (TAA) has been demonstrated with a related poxvirus-based immunotherapy expressing CEA. Those studies, performed in CEA-transgenic mice (where CEA is viewed by the immune system as a self-antigen) demonstrated, that anti-tumor efficacy following immunization with CEA/TRICOM vectors was largely due to treatment-induced T cell responses and occurred in the absence of autoimmunity [31]. Another study using PSA-transgenic mice, demonstrated that a single treatment with recombinant vaccinia virus expressing PSA could induce PSA-specific T cells and this response was augmented by androgen ablation [32]. Furthermore, preliminary data from one clinical study [33] revealed a significant decrease in Treg relative to CD4 positive cells within the tumor following intraprostatic administration of PROSTVAC-F. In other studies where PROSTVAC immunotherapy was given s.c. [6], there was a strong trend in changes in Treg function pre- versus post- PROSTVAC immunotherapy. Patients who survived longer than predicted by the Halabi nomogram had decreased Treg suppressive function after receiving PROSTVAC (log-rank P = 0.058).

Antigen spreading is the development of immune responses to TAA found on the tumor but not expressed by the poxvirus-based vector. Our data demonstrate that PROSTVAC immunotherapy induced antigen spreading from the originally targeted PSA to unknown antigens expressed by the parenteral mouse prostate cancer cell line. Importantly, evidence of antigen spreading has also been found in clinical studies where T cell responses to MUC-1, PSMA, PAP and other TAA have been detected in more than 50% of the analyzed patients [34-37]. Monitoring of these antigen spread responses may be of great value as an immune correlate for a successful anti-tumor response.

## Conclusions

In summary, we have generated preclinical evidence supporting the PROSTVAC mechanism of action and have identified potential immune correlates for anti-tumor efficacy using poxvirus-based immunotherapy in mice. These hypothesis-generating data will be applied to the retrospective and prospective analyses of clinical trials employing PROSTVAC and other poxvirus-based immunotherapies either alone or in combination with other therapies.

## Methods

### Viruses

The PROSTVAC vaccinia vector (PROSTVAC-V), fowlpox vector (PROSTVAC-F) and wild-type (WT) fowlpox (TBC-FPV) were produced by Bavarian Nordic (BN) at IDT Biologika (IDT, Dessau-Rosslau, Germany). A preparation of Dryvax was used for WT vaccinia virus (New York City Board of Health (NYCBH) Wyeth Strain, seed stock provided by the Centers for Disease Control and Prevention; manufactured by the former Therion Biologics Corp., Cambridge, MA). Fowlpox-muGM-CSF (rFP GM-CSF) is a recombinant fowlpox virus expressing mouse granulocyte-macrophage colony stimulating factor [16] and was produced by BN (Martinsried, Germany). The infectious unit titers (Inf.U/mL) of viral stocks were determined using flow cytometry [38]. Inactive virus particles were generated by exposing preparations to UV light for 20-40 minutes. The integrity of the inactivated virus particles was confirmed by Nanoparticle Tracking Analysis (data not shown; [39]).

### Proteins and peptides

The following peptides were synthesized by and purchased from JPT Peptide Technologies, Inc. (Acton, MA): PSA overlapping peptide library (PSA OPL), PSA-specific CD8 peptides (AQVHPQKVTKFMLCA and PQKVTKFMLCAGRWT for ELISPOT or HPQKVTKFML [11] for flow cytometry), PSA-specific CD4 peptides (PERPSLYTKVVHYRK and SLYTKVVHYRKWIKD), vaccinia E3L and F2L peptides (VGPSNSPTF and SPGAAGYDL [40]). The HER2-derived peptide p63 (TYLPTNASL [41]) was used as a negative control. PSA protein was purchased from Meridian Life Science®, Inc. (Memphis, TN).

### *In vivo* studies

Male BALB/c mice aged 6-8 weeks old were obtained from Simonsen Laboratories (Gilroy, CA). In non-tumor challenged studies, virus treatments were given subcutaneously (s.c.) on days 1, 15, and 29. Mice were dosed with either 2E6 Inf.U PROSTVAC-V, 1E7 Inf.U PROSTVAC-F or buffer as indicated in the results. Antibody titers were determined by ELISA, and the frequency of IFNγ producing T cells was determined by ELISPOT as described previously [42]. Animals received food and water *ad libitum* and were maintained according to BN IACUC guidelines.

For tumor challenge experiments, the mouse prostate cancer derived RM-11-PSA clone E6 [43] was expanded *in vitro* and 1.5E5 E6 cells were implanted intradermally (i.d.) in 100 μL DPBS on day 1. Mice were treated on days 1, 8, and 15 with 2E7/1E8 Inf.U WT vaccinia/WT fowlpox, 2E7/1E8 Inf.U PROSTVAC-V/F, 2E7/1E8 Inf.U PROSTVAC-V/F with 1E7 Inf.U of Fowlpox-muGM-CSF, or volumes equivalent to 2E7/1E8 Inf.U of UV-treated PROSTVAC-V/F. Tumor growth was measured twice a week using calipers. Tumor volume (mm^3^) was calculated using the formula: V = (LxW^2^)/2, L = length, W = width (1 mm^3^ = 1 mg). For each treatment group, mean tumor sizes were calculated. Error bars represent standard error of the mean (SEM). Statistical significance over the course of the measurements was determined by Repeated Measure ANOVA (RM-ANOVA) with a multiple comparison post-test (Tukey for 3 or more groups and Sidak for 2 groups). For depletion studies, mice were treated intraperitoneally (i.p.) on days -3, 5, 12, and 19 with anti-CD4 (GK1.5), anti-CD8 (2.43) or isotype control antibodies from BioXCell (West Lebanon, NH).

### Flow cytometry

Spleens and solid tumors were collected for flow cytometric analysis. Cell suspensions of the spleen were prepared by gently grinding the tissue between the frosted ends of glass microscope slides in RPMI-10: RPMI-1640 (Corning, Manassas, VA), 10% FBS (Corning), 1% penicillin/streptomycin (Corning), and 0.55 μM β-Mercaptoethanol (Life Technologies, Carlsbad, CA). Splenocytes were treated with ACK lysing buffer (Lonza, Allendale, NJ). Solid tumors were diced to ~1-2 mm^3^ pieces and further digested to single cell suspensions for 1 h at 37°C in RPMI-10 with 50 U/mL DNAse I and 250 U/mL Collagenase I (Worthington Biochemical Corporation, Lakewood, NJ). Red blood cells were lysed with RBC Lysis Buffer (eBioscience, San Diego, CA).

Antibodies used for flow cytometry (clone number in parentheses): CD3 (500A2), CD4 (RM4-5), CD8a (53-6.7), CD107a (1D4B), KLRG1 (2 F1), CD44 (clone IM7), CD62L (MEL-14), and IFNγ (XMG1.2), from BD Biosciences (San Jose, CA). CD3 (145-2C11), CD127 (A7R34), CD4 (RM4-5), CD8 (53-6.7), IL-2 (JES6-5H4), IFNγ (XMG1.2), and TNFα (MP6-XT22) from BioLegend (San Diego, CA). CD4 (RM4-5), ICOS (7E.17G9), FoxP3 (FJK-16 s) and FC receptor block CD16/32 (93) from eBioscience. Flow cytometry was performed on the LSR II (BD Biosciences) and analyzed using FlowJo version 9 (TreeStar, Ashland, OR).

To characterize antigen-specific cytotoxic T lymphocytes (CTL), an activation-induced degranulation assay was used [44] to identify CD107a^+^ IFNγ^+^ CD8 T cells. Splenocytes were plated at 2E6 cells per well in RPMI-10 and cells were stimulated overnight at 37°C with antigen-specific peptides, 1 μM human p63 (an irrelevant peptide control), 5 μg/mL Concanavalin A (MP Biomedicals, Santa Ana, CA), or RPMI-10 in the presence of anti-CD107a and GolgiStop™ (BD Biosciences). The following day, the cells were washed, blocked with FC-block and stained for surface markers. Cells were then stained intracellularly for IFNγ using the BD Cytofix/Cytoperm™ kit (BD Biosciences). Additional intracellular cytokine staining was performed on splenocytes to detect IFNγ, TNFα, IL-2, and IL-4. Cells were stimulated overnight with antigen-specific peptides, 1 μM human p63 as negative control, 2 nM PMA/1 μM ionomycin (Acros Organics, Lawndale, NJ and Sigma-Aldrich, St. Louis, MO) or RPMI-10 in the presence of GolgiStop™ and Golgi Plug™ (BD Biosciences) and stained as previously described.

Regulatory T cells were identified by staining intracellularly for FoxP3 using the FoxP3/Transcription Factor Staining Buffer Set from eBioscience following the manufacturer’s instructions.

## Additional file

Additional file 1:
**Supplemental Data.**
