# Supplementary material for: Elucidating immunologic mechanisms of PROSTVAC cancer immunotherapy
Source: J Immunother Cancer. 2014 Oct 14;2:34. doi: 10.1186/s40425-014-0034-0 (PMC4201731; doi:10.1186/s40425-014-0034-0)
Supplement: Additional file 1: — Supplemental Data. [file 40425_2014_34_MOESM1_ESM.pdf]

Supplemental Data:

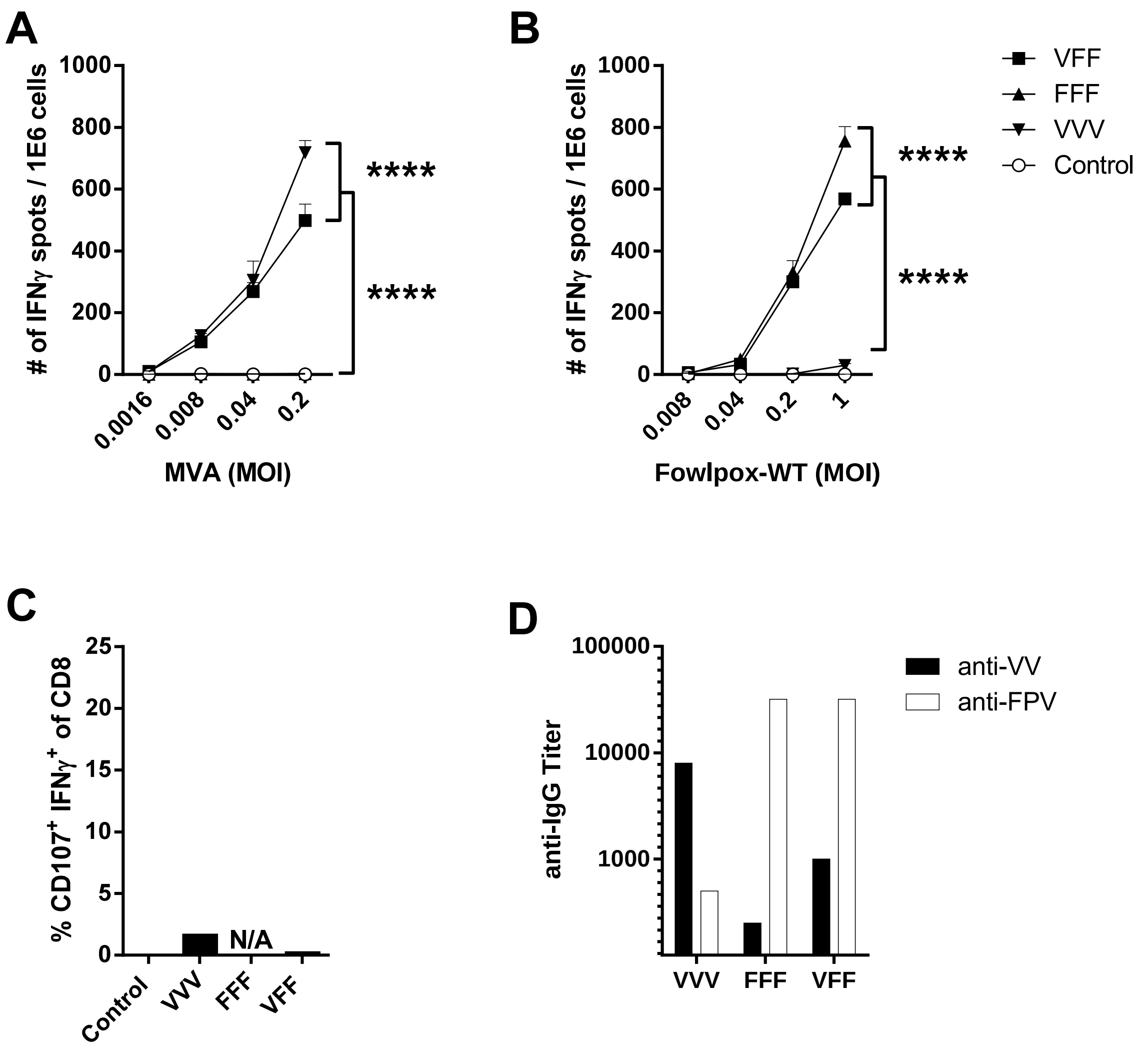

Figure SD1: Vector-specific T cell and antibody responses. BALB/c males (5/group) were treated every two weeks with: Buffer (Control), PROSTVAC-V (VVV), PROSTVAC-F (FFF) or received a PROSTVAC-V prime followed by 2 PROSTVAC-F boosts (VFF). Pooled splenocytes were assayed for vector-specific responses by IFNγ ELISPOT (A, B) and cytotoxic activity by flow cytometry (% $CD107^{+}$ $IFN\gamma^{+}$ CD8 T cells) (C). Due to the lack of an appropriate reagent, cytotoxic activity against the vector could only be determined for vaccinia virus. Vector-specific antibody titer were only determined for pooled samples at the end of study (day 42) (D). For ELISPOT, splenocytes were restimulated with Modified Vaccinia Ankara (MVA) or WT fowlpox virus at indicated multiplicity of infection (MOI). Statistical significance was determined by Two-Way-ANOVA with Tukey post-test. ****P < 0.001 at MOI 0.2 for MVA and MOI 1 for Fowlpox-WT compared to control or homologous dosing (A & B). To identify cytotoxic CD8+ T cells, splenocytes were restimulated overnight with vaccinia-specific CD8 peptides (E3L and F2L see material and methods) in the

presence of anti-CD107 antibody. Graphs show representative data of four independently performed experiments.

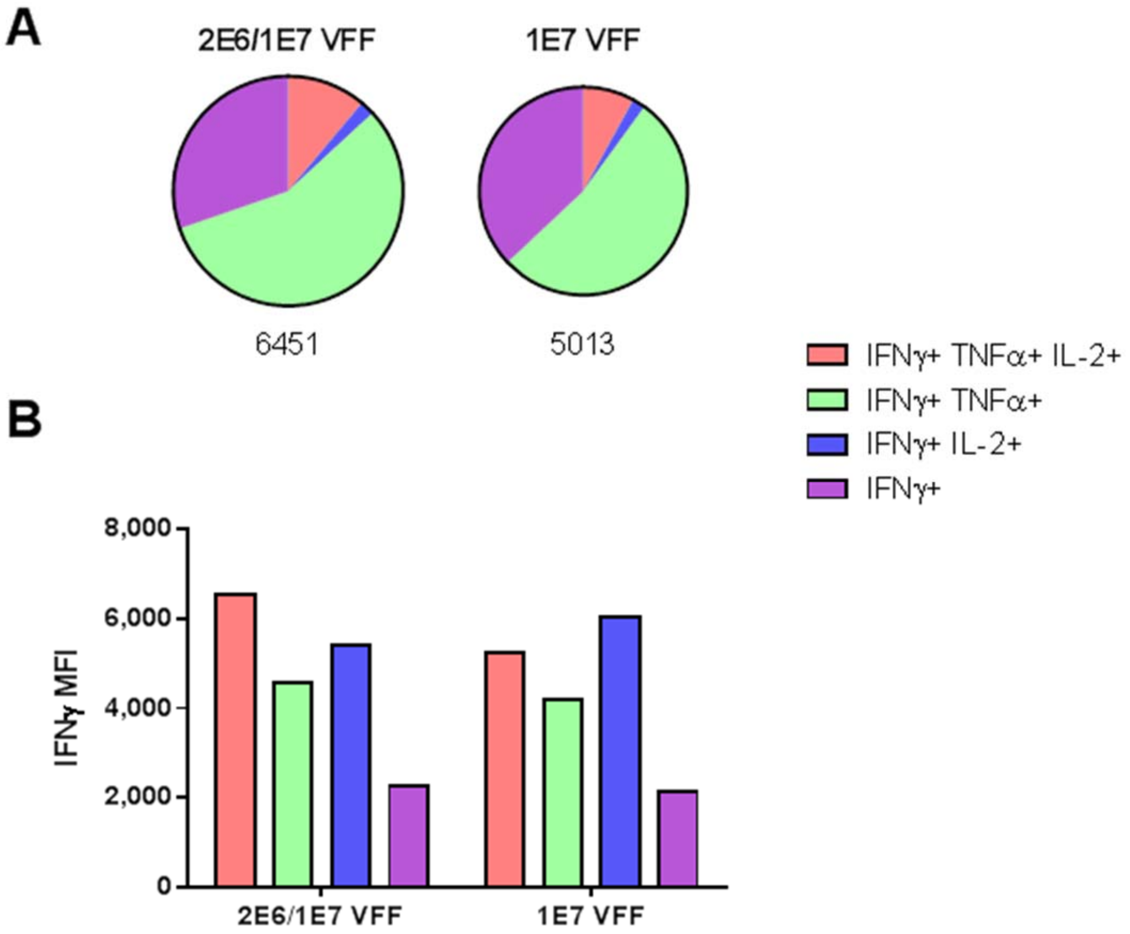

Figure SD2: Priming with a 5-fold higher PROSTVAC-V dose did not yield any additional benefit in the magnitude or the quality of the CD8 T cell response. BALB/c mice (6/group) were treated as described for Figure 1. Spleens were harvested 14 days after the last treatment, and pooled splenocytes were restimulated overnight with PSA OPL (controls consisted of irrelevant OPL of similar library size, data not shown). The cells were stained for intracellular IFNγ, TNFα, and IL-2, prior to flow cytometric analysis. (A) The pie charts are weighted in size to reflect the numbers of detected cells (total numbers of PSA-specific CD8 T cells per million T cells are indicated below each pie). (B) Amount of IFNγ production on a per cell basis as measured by mean fluorescence intensity (MFI). Graphs show representative data of two independently performed experiments.

SD Table1: Relative % of T cell subpopulations described in Figures 2A and SD2 (pie charts)

	2E6 VVV	1E7 FFF	2E6/1E7 VFF	1E7 VFF
IFNγ+ TNFα+ IL-2+	15%	3%	11%	8%
IFNγ+ TNFα+	25%	43%	56%	53%
IFNγ+ IL-2+	10%	2%	2%	2%
IFNγ+	50%	51%	30%	37%
	2E6 VVV	1E7 FFF	2E6/1E7 VFF	1E7 VFF
Sum of TNFα triple and double positive	40%	46%	67%	61%

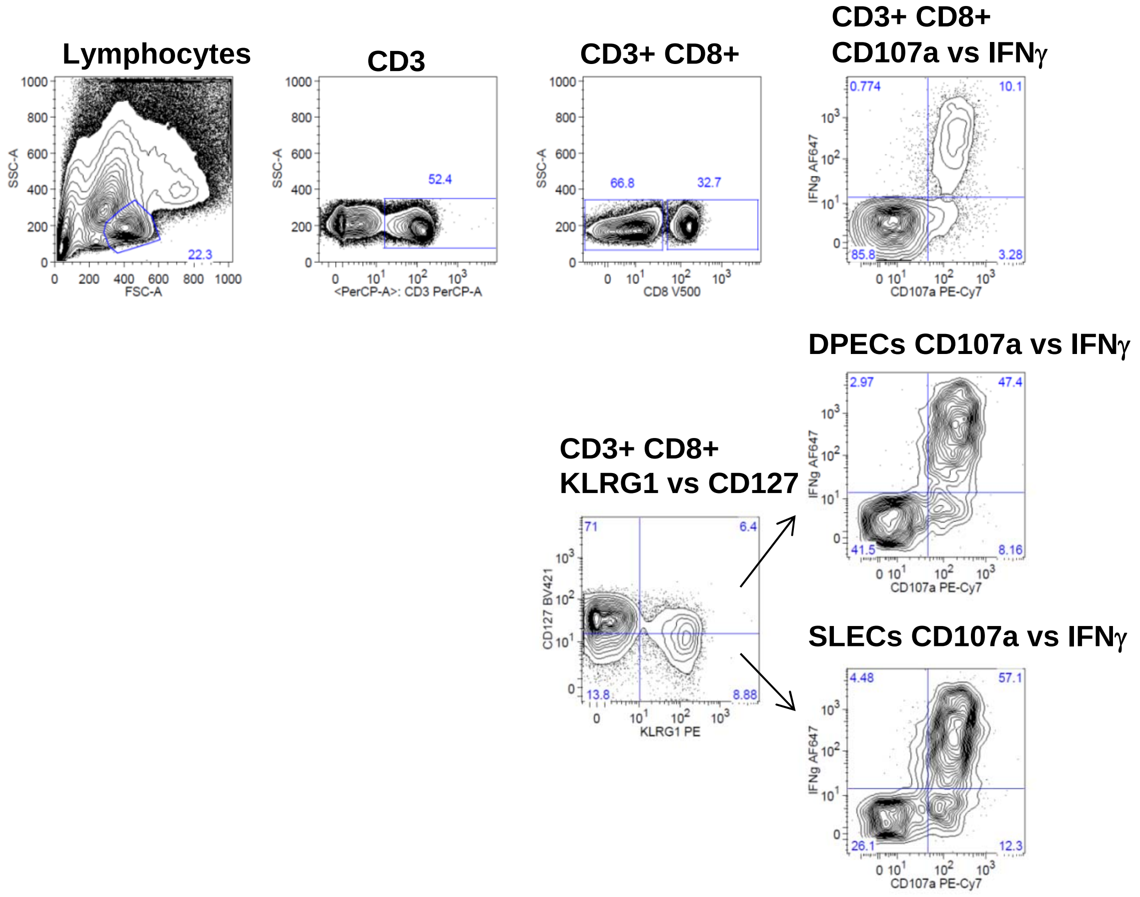

Figure SD3: Gating strategy for Figure 3. Lymphocytes were restimulated with PSA or VV-specific peptides overnight in the presence of CD107 antibody. The next day, cells were stained intracellularly for IFNγ and for the indicated surface markers. Gates were determined with the use of fluorescence minus one controls (FMOs), which was especially useful for setting the CD127 gate due to the lack of distinct positive and negative populations. Graphs show representative data of three independently performed experiments.

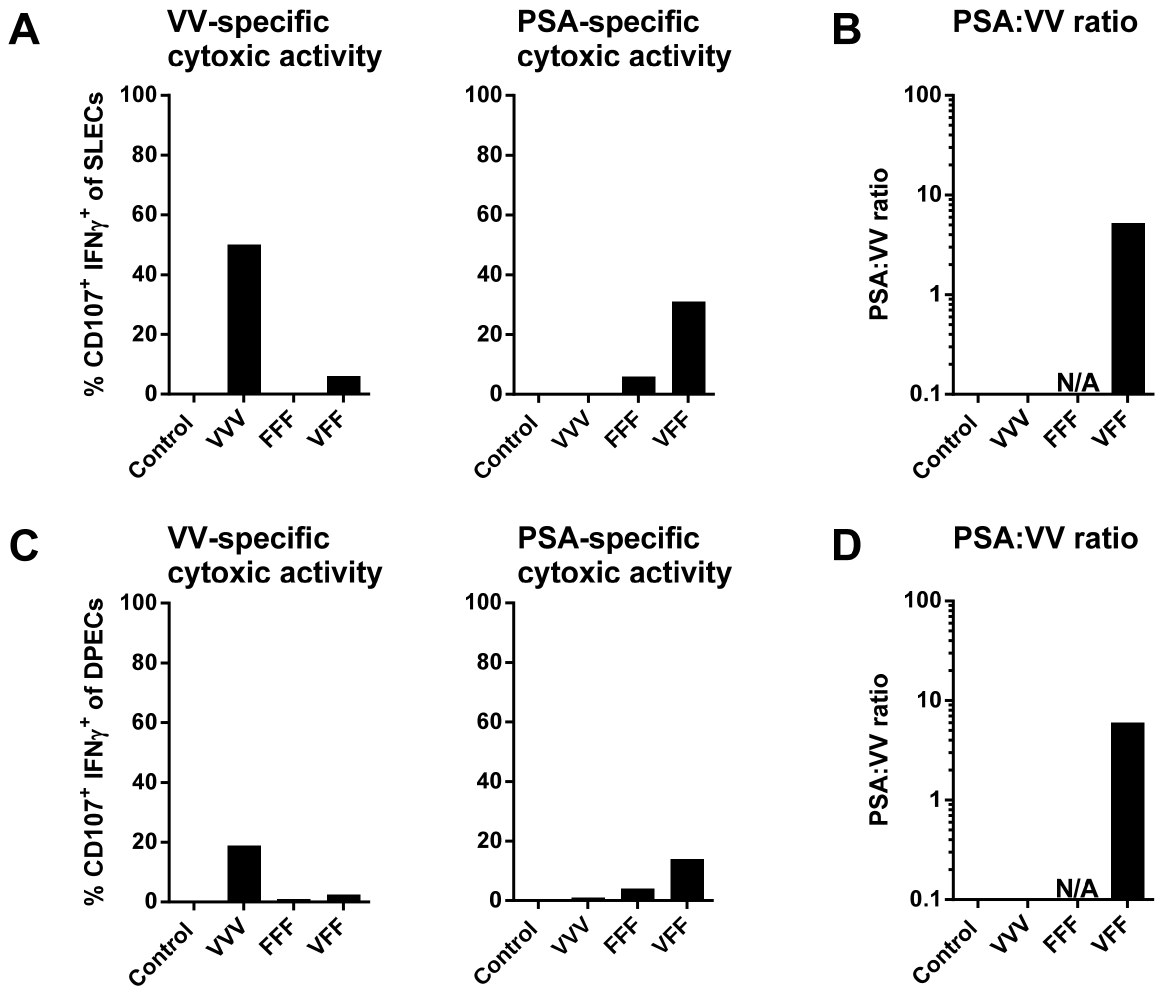

Figure SD4: Immune focusing of T cell response towards PSA

Mice were treated as described for Figure 1. Pooled splenocytes were assayed for vaccinia virus (VV)-specific ((A) and (C) panels on left) or PSA-specific ((A) and (C) panels on right) cytotoxic activity by flow cytometry (% CD107+ IFNγ+ CD8 T cells) 14 days after the last treatment. Splenocytes were restimulated overnight with vaccinia E3L and F2L peptides or with PSA peptide HPQKVTKFML in the presence of anti-CD107 antibody. The following day, cells were stained intracellularly for IFNγ and with the surface markers CD127 and KLRG1. % antigen-specific cytotoxic SLEC and DPEC were determined by gating on (CD8+CD127-KLRG1+) and (CD8+CD127+KLRG1+) cells, respectively. Graphs show representative data of two independently performed experiments. The PSA to VV ratio was determined by dividing %CD107+ IFNγ+ PSA-specific DPECs or SLECs by %CD107+ IFNγ+ VV-specific DPECs or SLECs. Graphs show representative data of two independently performed experiments.

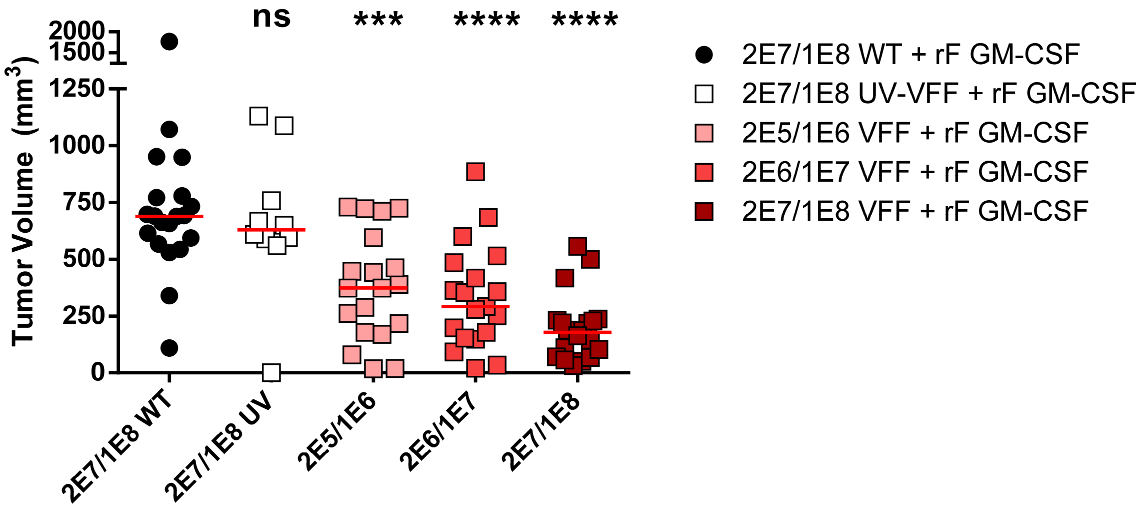

Figure SD5: PROSTVAC mediated anti-tumor efficacy over a 100-fold dose range. BALB/c mice (10-20/group) were challenged by i.d. injection of RM-11-PSA cells on day 1 and treated 3 times at weekly intervals as described in text. Statistical significance determined at day 16 (One-Way ANOVA with Bonferroni's multiple comparison post-test).***p<0.001, ****p<0.0001. Graphs show representative data of two independently performed experiments.

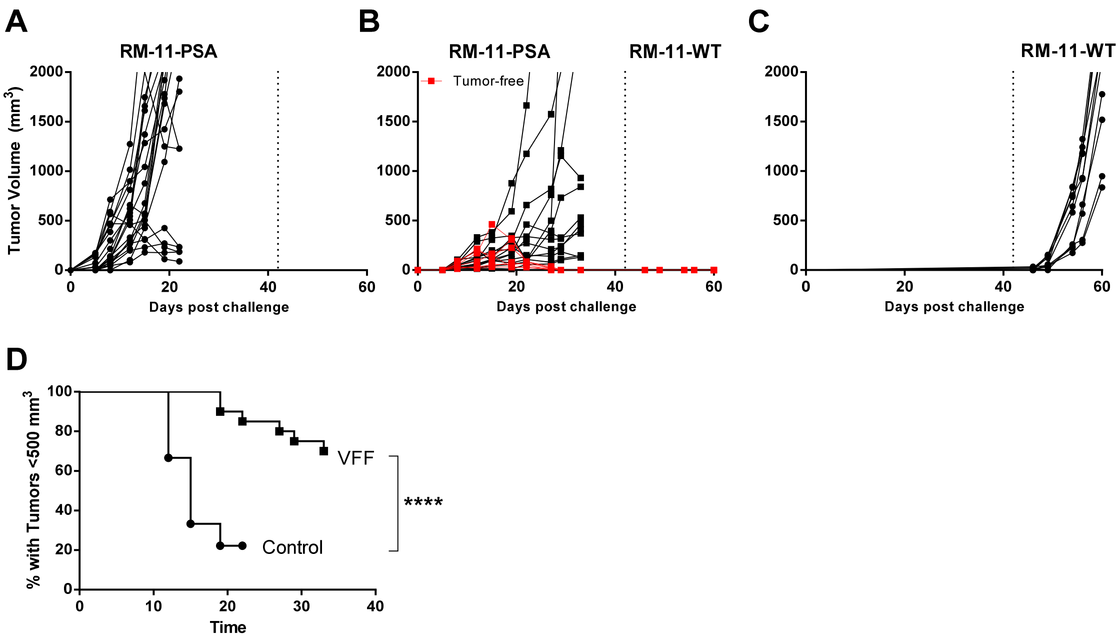

Figure SD6: PROSTVAC mediated anti-tumor efficacy and antigen spreading (A&B) BALB/c mice were challenged by i.d. injection of RM-11-PSA cells on day 1 and treated 3 times at weekly intervals (d1, 8, 15) with Buffer ((A) n=20) or PROSTVAC V/F ((B) n=20). All control treated mice were sacrificed on day 23. VFF treated mice with tumor were taken down on day 33, 6 tumor free mice were kept for re-challenge with WT cells. (D) Kaplan-Meier curves were generated based upon a tumor volume of 500 mm^3. (B&C) On day 42 (dotted line), tumor-free PROSTVAC-V/F treated mice (in red n=6) and naïve BALB/c mice ((C) n=10) were injected i.d. with 2E5 RM11-WT cells (these cells do not express PSA). Statistical analysis determined by Mantel-Cox and Gehan-Breslow-Wilcoxon tests. ****p<0.0001

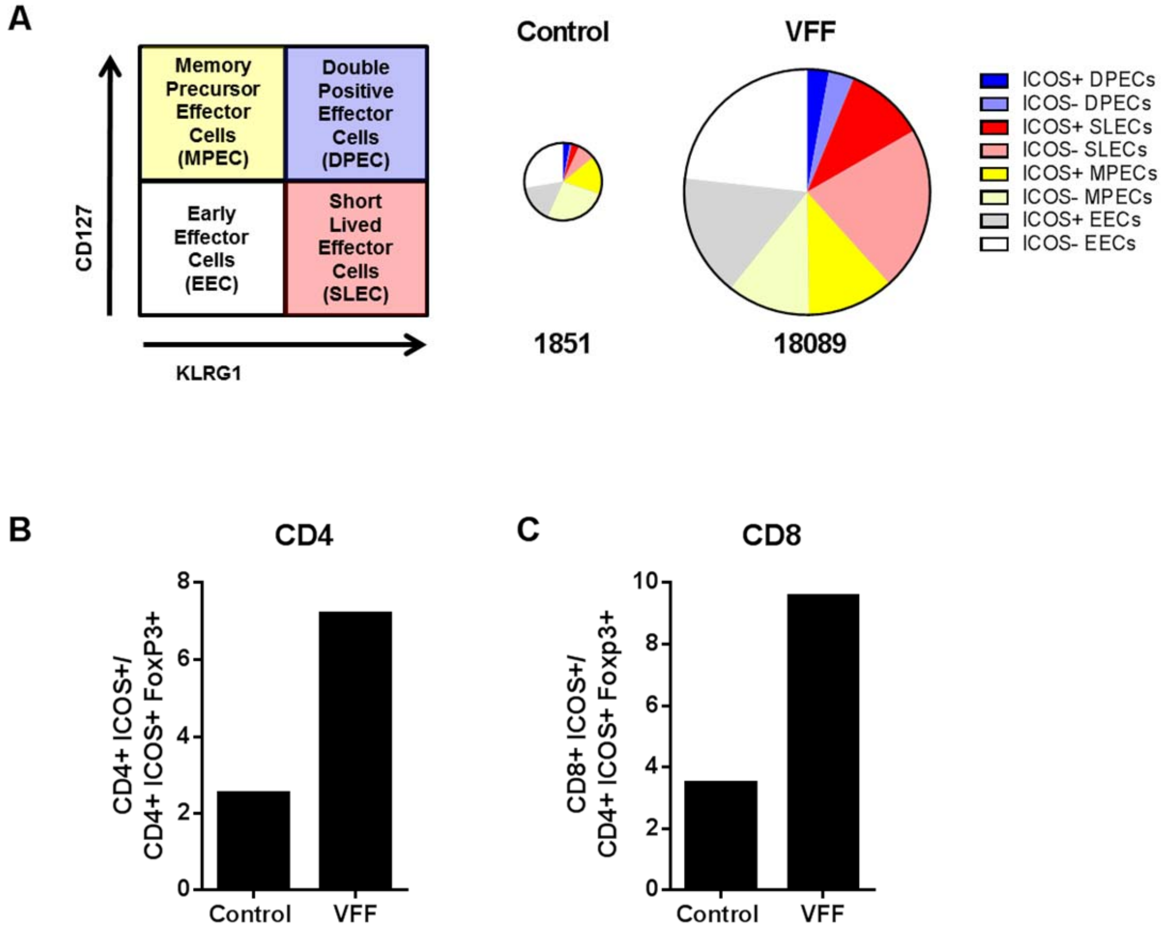

Figure SD7: PROSTVAC immunotherapy expands highly activated ICOS+ effector T cells in the tumor and improves the ratio of activated T_{eff}:T_{reg} cell ratios. **(**A) Schematic for the characterization of effector and memory cells. Pie charts are weighted in size to reflect the numbers of detected cells (numbers below indicate total number of CD8 T cells/million tumor cells). (B) CD4 and (C) CD8 activated T_{eff}:T_{reg} ratios were calculated by dividing the number of CD4+ ICOS+ or CD8+ ICOS+ T cells by the number of CD4+ ICOS+ FoxP3+ cells. Graphs show representative data of four independently performed experiments.
